# Clinical Significance of ARID1A and ANXA1 in HER-2 Positive Breast Cancer

**DOI:** 10.3390/jcm9123911

**Published:** 2020-12-02

**Authors:** Rita Silva-Oliveira, Filipa Ferreira Pereira, Sara Petronilho, Ana Teresa Martins, Ana Lameirinhas, Vera Constâncio, Inês Caldas-Ribeiro, Sofia Salta, Paula Lopes, Luís Antunes, Fernando Castro, Susana Palma de Sousa, Rui Henrique, Carmen Jerónimo

**Affiliations:** 1 Cancer Biology & Epigenetics Group—Research Center, Portuguese Oncology Institute of Porto (CI-IPOP), Rua Dr. António Bernardino de Almeida, 4200-072 Porto, Portugal; rita.silva.oliveira@ipoporto.min-saude.pt (R.S.-O.); sara.petronilho@ipoporto.min-saude.pt (S.P.); atmartins@ipoporto.min-saude.pt (A.T.M.); ana.lameirinhas@ipoporto.min-saude.pt (A.L.); vera.salvado.constancio@ipoporto.min-saude.pt (V.C.); ines.c.ribeiroo@gmail.com (I.C.-R.); sofia.salta@ipoporto.min-saude.pt (S.S.); ana.ambrosio@ipoporto.min-saude.pt (P.L.); rmhenrique@icbas.up.pt (R.H.); 2 Institute of Biomedical Sciences Abel Salazar, University of Porto (ICBAS-UP), Rua de Jorge Viterbo Ferreira n. 228, 4050-313 Porto, Portugal; 3 Breast Cancer Clinic and Department of Medical Oncology, Portuguese Oncology Institute of Porto, Rua Dr. António Bernardino de Almeida, 4200-072 Porto, Portugal; filipa.pereira@ipoporto.min-saude.pt (F.F.P.); susana.sousa@ipoporto.min-saude.pt (S.P.d.S.); 4 Department of Pathology, Portuguese Oncology Institute of Porto, Rua Dr. António Bernardino de Almeida, 4200-072 Porto, Portugal; 5 Cancer Epidemiology Group—Research Center & Department of Epidemiology, Portuguese Oncology Institute of Porto, Rua Dr. António Bernardino de Almeida, 4200-072 Porto, Portugal; luis.antunes@ipoporto.min-saude.pt; 6 Breast Cancer Clinic and Department of Surgical Oncology, Portuguese Oncology Institute of Porto, Rua Dr. António Bernardino de Almeida, 4200-072 Porto, Portugal; fernando.castro@sapo.pt; 7 Department of Pathology and Molecular Immunology, Institute of Biomedical Sciences Abel Salazar, University of Porto (ICBAS-UP), Rua de Jorge Viterbo Ferreira n. 228, 4050-313 Porto, Portugal

**Keywords:** ANXA1, ARID1A, biomarkers, breast cancer, trastuzumab

## Abstract

Background: trastuzumab is considered the standard of care for human epidermal growth factor receptor-2 (HER-2+) breast cancer patients. Regardless of the benefits of its use, many early-stage patients eventually recur, and usually, the disease progresses within a year. Since about half of the HER-2+ patients do not respond to trastuzumab, new biomarkers of prognosis and prediction are warranted to allow a better patient stratification. Annexin A1 (ANXA1) was previously reported to contribute to trastuzumab resistance through AKT activation. An association between adenine thymine-rich interactive domain 1A (ARID1A) loss and ANXA1 upregulation was also previously suggested by others. Methods: in this study, we examined tissue samples from 215 HER-2+ breast cancer patients to investigate the value of ARID1A and ANXA1 protein levels in trastuzumab response prediction and patient outcome. Expression of ARID1A and ANXA1 were assessed by immunohistochemistry. Results: contrary to what was expected, no inverse association was found between ARID1A and ANXA1 expression. HER-2+ (non-luminal) tumours displayed higher ANXA1 expression than luminal B-like (HER-2+) tumours. Concerning trastuzumab resistance, ARID1A and ANXA1 proteins did not demonstrate predictive value as biomarkers. Nevertheless, an association was depicted between ANXA1 expression and breast cancer mortality and relapse. Conclusions: overall, our results suggest that ANXA1 may be a useful prognostic marker in HER-2+ patients. Additionally, its ability to discriminate between HER-2+ (non-luminal) and luminal B-like (HER-2+) patients might assist in patient stratification regarding treatment strategy.

## 1. Introduction

Breast cancer (BrC) is the most common cancer and the leading cause of cancer-related death among women, being responsible for high morbidity and mortality rates. This constitutes as a health issue and economic burden worldwide [1].

Currently, in clinical practices, different molecular subtypes are recognized based on a panel of immunohistochemical biomarkers, including oestrogen receptors (ER) and progesterone receptors (PR), and the human epidermal growth factor receptor-2 (HER-2) [2,3]. Although the majority of diagnosed tumours express ER and PR, about 20–30% of cases express the hormonal receptors and HER-2, and are classified as luminal B-like (HER-2 positive), whereas those named HER-2 positive (non-luminal) only overexpress HER-2 [2,3,4,5]. Despite having different prognosis and treatment strategies, patients with luminal B-like (HER-2 positive) and HER-2 positive (non-luminal) BrC receive trastuzumab therapy [5].

Trastuzumab is a monoclonal antibody directed to the extracellular domain of the HER-2 protein [3]. It was approved in 1998 for the treatment of HER-2 positive (HER-2+) metastatic BrC (MBC), having revolutionized the precision medicine for this disease and becoming a standard of care in the HER-2+ disease [6]. Indeed, according to the European Society for Medical Oncology (ESMO) guidelines, when combined with chemotherapy, trastuzumab halves the recurrence and mortality risks, compared with chemotherapy alone. Although being usually well-tolerated, patients may experience cardiac dysfunction, which is further exacerbated when administered concomitantly with anthracyclines [5].

Nevertheless, resistance to trastuzumab occurs in approximately 20% of early-stage BrC and 70% of MBC, and so a need to understand the mechanisms underlying this lack of response is a major concern [7]. Moreover, apart from HER-2 overexpression, there is still no reliable trastuzumab predictive biomarker, which translates into the current inability to effectively stratify patients. This highlights the need to find new predictive biomarkers that can help in this task [8].

Upregulation of the phosphatidylinositol 3-kinases/protein kinase B/mammalian target of rapamycin (Pi3K/AKT/mTOR) pathway has been associated with resistance to drugs that target the HER kinases [8,9,10,11,12]. This pathway is one of the HER-2 downstream signalling pathways and its activation leads to the prevention of trastuzumab-mediated growth arrest in HER-2 amplified BrC cells through increased cell motility, survival, and proliferation [8,12].

In 2016, a report by Berns and colleagues established a functional relationship between adenine thymine-rich interactive domain 1A (ARID1A) and annexin A1(ANXA1) [13]. Specifically, ARID1A loss was correlated with ANXA1 upregulation, which, in turn, activates the Pi3k/AKT/mTOR pathway leading to trastuzumab resistance, suggesting that HER-2+ BrC patients with high ANXA1 expression are less likely to respond to trastuzumab therapy.

ARID1A gene encodes a nuclear protein (BAF250a), which is a subunit of the SWI/SNF chromatin remodelling complex, a critical regulator of differentiation, proliferation, DNA repair, and tumour suppression [14,15,16]. Moreover, a tumour suppressor function has been established for this gene in endometrial, cervical, lung, and renal cancers [17,18,19,20].

ANXA1 is a calcium and phospholipid-binding protein, firstly reported to have anti-inflammatory activity. Additional studies have implicated ANXA1 in other significant cellular mechanisms such as signal transduction, cell survival, proliferation, differentiation, migration, and disease development [21,22,23]. Some studies have already addressed ANXA1 involvement in the development and progression of some types of cancers and also in therapy resistance in in vitro assays [23]. Nonetheless, ANXA1 was shown to be downregulated in prostate, oesophageal, and cervical cancers, and upregulated in liver, colorectal, and lung cancers, among others. In breast cancer, both tumour suppressor and oncogenic activity have been attributed to ANXA1 [23].

Nevertheless, ARID1A and ANXA1’s role in HER-2+ BrC prognosis and, specifically, in resistance to trastuzumab, is still poorly understood. Moreover, the relationship between the two genes has not yet been properly addressed. Therefore, we aimed to investigate their value as prognostic or predictive biomarkers in HER-2+ BrC.

## 2. Experimental Section

### 2.1. Patients and Samples Collection

The study retrospective cohort comprises 215 consecutive HER-2+ BrC patients, diagnosed from 2008 to 2013 and treated with a trastuzumab-based therapy at the Portuguese Oncology Institute of Porto, Portugal (IPO-PORTO). Formalin-fixed paraffin-embedded (FFPE) tissue samples collected before patients’ treatment, available in the Department of Pathology archives, were analysed. 

Haematoxylin and eosin (H&E) slides were reviewed by an experienced pathologist according to the World Health Organization (WHO, France) classification [24]. Relevant clinical data was collected from clinical records (until May of 2019, median follow-up: 83 months) and displayed in an anonymized database for analysis purposes.

This study was approved by the institutional review board (Comissão de Ética para a Saúde-CES 125/019) of IPO-PORTO. 

### 2.2. Immunohistochemistry (IHC)

Briefly, tumour blocks were sectioned at a thickness of 4 µm, deparaffinised in xylene, and hydrated through a graded alcohol series. Antigen retrieval was achieved by microwave or water bath for 20 min in ethylenediaminetetraacetic acid (EDTA) buffer. Endogenous peroxidases were inactivated by 0.6% hydrogen peroxide (H_2_O_2_) and blocking of antibody nonspecific binding was achieved through incubation with horse serum (Vector Laboratories S-2000 Normal Horse Serum, concentrated; 20 mL) in a 1:50 dilution for 20 min. Slides were then incubated, according to optimized conditions, with each primary antibody (Table 1). Normal oesophagus tissue was used as an external positive control for ANXA1 and normal cervix for ARID1A antibody.

The slides were incubated with a post-primary block and then with polymer (Novocastra Novolink™) for 30 min each. Following, diaminobenzidine tetrahydrochloride (DAB), diluted in phosphate-buffered saline (PBS), was used as a chromogen. Lastly, the slides were counterstained with haematoxylin. 

### 2.3. Immunohistochemistry Scoring

In each case, lymphocytes were used as an internal positive control, for the evaluation of both antibodies. 

For ANXA1 immunostaining, the percentage of cells stained and cytoplasmic intensity of staining were assessed. The intensity was scored from 0 to +3, representing negative to strong staining. A score of +3 was assigned when the intensity of staining was equivalent to that of lymphocytes. The overall score was determined as previously described [25]: *Overall score = ((%cells with intensity score 1) × 1)) + ((%cells with intensity score 2) × 2)) + ((%cells with intensity score 3) × 3))*.

Concerning ARID1A, since all samples had approximately 90–100% of cells stained, only intensity was evaluated. Intensity ranged from score 0 (absence), 1+, 2+, which was equivalent to lymphocytes staining intensity, and 3+, indicative of high intensity. 

For the statistical analysis, as no clear cut-off was defined in the literature, ANXA1 staining was categorized into “negative” (73.9%) and “positive” (26.1%) expression, considering the 75 percentile. On the other hand, ARID1A was grouped into two categories: “low intensity” (93.8%), comprising intensity scores 1+ and 2+, and “high intensity” (6.2%) comprising only the 3+ intensity score. 

### 2.4. Statistical Analysis

All data were analysed using SPSS statistical software (version 24.0, Chicago, IL, USA) and R software (version 3.4.4, Vienna, Austria). Non-parametric tests were used to compare ARID1A and ANXA1 immunoexpression between molecular subtypes and to evaluate associations with other clinicopathological features. Differences in protein immunoexpression between molecular subtypes and other clinicopathological variables were assessed by Pearson’s chi-square or Fisher’s exact test. 

Cumulative incidence of breast cancer mortality (CIBCM), cumulative incidence of relapse (CIR), and cumulative incidence of trastuzumab resistance relapse (CITRR) curves were assessed through a competing risk method, and Gray’s test was used to test differences between groups. Univariable Cox regression was used to assess standard clinicopathological variables and proteins’ prognostic value. To understand which variables remained independent predictors of mortality, a multivariable analysis was performed using the Cox proportional hazards model using the backward conditional method. A *p*-value of less than 0.05 was considered statistically significant. 

CIR was defined as the time between surgery date and recurrence date and CIBCM was defined as the time between diagnostic date and death from the disease. To perform CITRR analysis, a new variable called “resistant” was created. Patients who presented disease recurrence during trastuzumab therapy, or up to 6 months after cessation, were considered resistant. 

All graphs were constructed using GraphPad Prism version 6.01 for Windows (GraphPad Software, La Jolla, CA, USA). 

## 3. Results

### 3.1. Clinical and Pathological Data

This study comprised 215 female patients with HER-2+ BrC, whose treatment included trastuzumab (Table 2). Seventy-eight percent of tumours were luminal B-like (HER-2+) and 22% were HER-2+ (non-luminal), as assessed by IHC assay. Most of the tumours were invasive carcinomas of no special type (NST), grade (G) 3, and stage I/II.

### 3.2. Resistance to Trastuzumab

**Figure 1:**
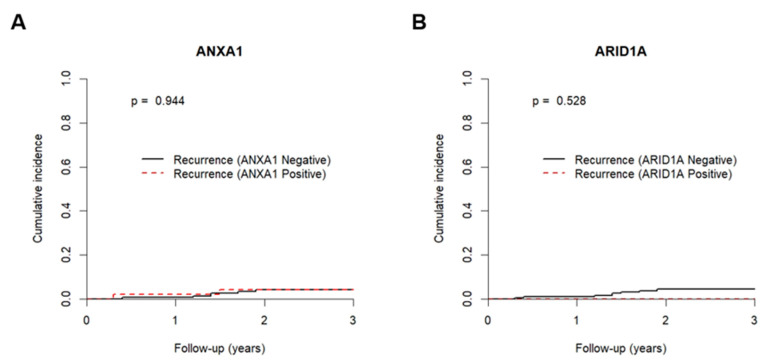
Cumulative incidence function plots according to (**A**) ANXA1 and (**B**) ARID1A immunoexpression. Dashed red line and full black line represent positive/high expression and negative/low expression for ANXA1 and ARID1A immunoexpression, respectively. *p* values obtained by Gray’s test for trastuzumab resistance relapse.

The value of ARID1A and ANXA1 expression as predictive biomarkers of trastuzumab resistance was assessed using disease recurrence as an endpoint. Therefore, patients who showed radiological evidence of disease during treatment with trastuzumab or within 6 months after trastuzumab cessation were considered resistant [26]. Since our cohort was comprised of two distinct molecular subtypes, we assessed if luminal B-like (HER-2+) and HER-2+ (non-luminal) displayed different survival, but no differences were depicted. Furthermore, since the small number of HER-2+ (non-luminal) patients (*n* = 48) would compromise the statistical power, cumulative incidence analysis was performed for all patients and not stratified by molecular subtype. Only nine patients presented recurrent tumours within this time period, and neither ARID1A nor ANXA1 expression predicted trastuzumab resistance in this group of BrC patients (Figure 1). 

### 3.3. Relationship between ARID1A and ANXA1

**Figure 2:**
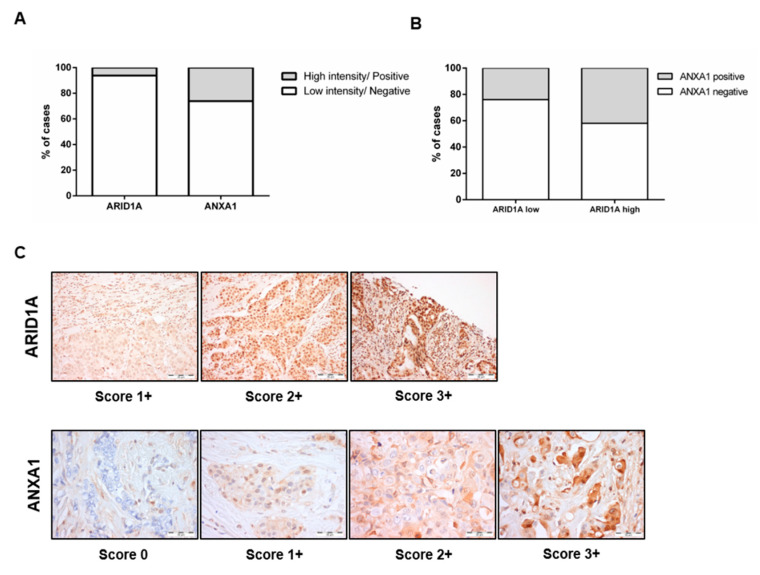
Relationship between ARID1A and ANXA1. Proportion of tumours with ARID1A high or low intensity and ANXA1 negativity or positivity (**A**). Association between ARID1A and ANXA1 (Chi-square: *p* = 0.183) (**B**). Illustrative images of the different protein intensity scores (**C**).

Contrary to what was previously reported [13], no inverse association was found between ANXA1 and ARID1A immunoexpression (Chi-square: *p* = 0.183) (Figure 2). On the contrary, tumours with ANXA1 expression seem to also exhibit higher ARID1A protein levels (Figure 2B).

### 3.4. ANXA1 Expression Is Higher in the HER-2+ (Non-Luminal) Subtype

**Figure 3:**
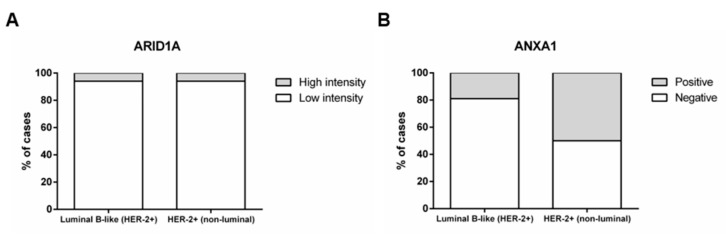
Distribution of ARID1A and ANXA1 immunoexpression by molecular subtype. Percentage of cases with low and high ARID1A intensity staining score (1+ and 2+ vs. 3+) (Chi-square *p* = 0.749) (**A**). Percentage of cases with or without ANXA1 expression (Chi-square *p* < 0.001) (**B**).

Although ARID1A expression did not associate with BrC molecular subtype, HER-2+ (non-luminal) tumours depict higher ANXA1 protein levels (*p* < 0.001) than luminal B-like (HER-2+) tumours (Figure 3).

No significant associations were found between both ANXA1 and ARID1A protein immunoexpression, and any other clinicopathological variable (age, histological type, lymphovascular invasion, grade, T stage, N stage, and stage). 

### 3.5. High ARID1A and ANXA1 Expression Is Associated with Early Recurrence and High Mortality 

Patients’ median follow-up time was 83 months. From the 215 patients included in this study, 31 (14.4%) deceased due to BrC, whereas 180 stayed alive, 10 of which (4.7%) harbouring cancer.

Due to the reduced number of events and/or cases in some categories, some clinicopathologic features were grouped. The grade was grouped as (G1 and G2 vs. G3), T stage as (T1 and T2 vs. T3 and T4), N stage as (N0 vs. N1), and stage as (I and II vs. III). ANXA1 was grouped as “negative vs. positive” according to p75 final score, whereas ARID1A protein staining intensity was grouped as “1+ and 2+ vs. 3+”, as previously stated.

Since the small number of HER-2+ (non-luminal) patients could compromise statistical analysis, cumulative incidence analysis was performed for the whole cohort and not stratified by molecular subtype.

**Figure 4:**
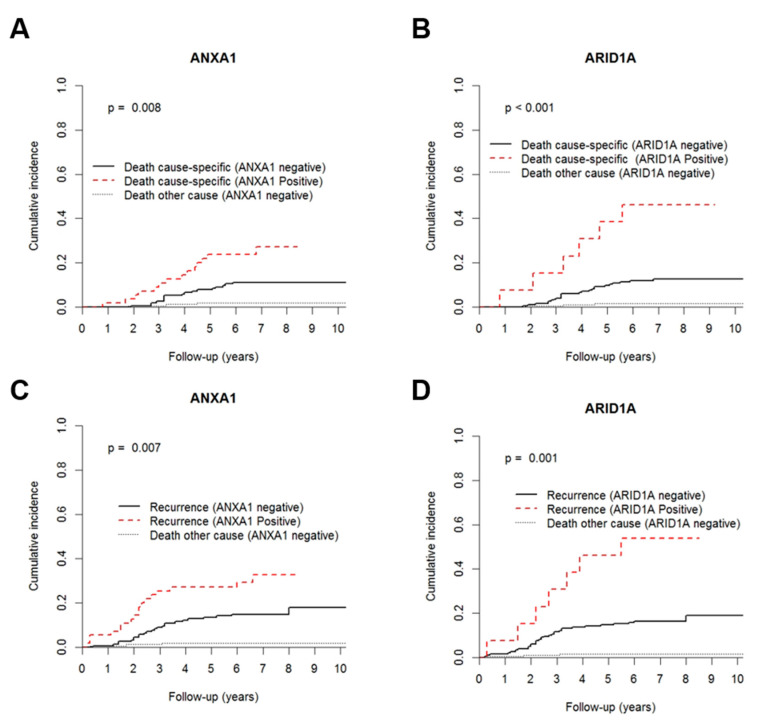
Cumulative incidence function plots according to (**A**,**C**) ANXA1 and (**B**,**D**) ARID1A immunoexpression. Dashed red line and full black line represent positive/high expression and negative/low expression for ANXA1 and ARID1A immunoexpression, respectively. *p* values obtained by Gray’s test for breast cancer mortality (**A**,**B**) and relapse (**C**,**D**).

Lymphovascular invasion, larger tumours (T3 and T4), positive lymph node, and clinical stage III at diagnosis significantly associated with an increased cumulative incidence of BCM and recurrent disease (Supplementary File 1—Figures S1 and S2). Notably, both CIBCM and CIR were significantly increased in patients with higher ANXA1 and ARID1A levels (Figure 4).

Despite disclosing shorter CIBCM and CIR, lymphovascular invasion, T stage, N stage, and ARID1A were not included in the Cox regression analysis due to the reduced number of events in each group. Remarkably, in the multivariable analysis, along with stage, ANXA1 immunoexpression was found to be an independent BrC mortality predictor (Table 3).

Strikingly, patients with ANXA1 positive tumours have, approximately, three times more probability of dying from BrC than those without expression. Additionally, ANXA1 positivity independently predicted shorter time to recurrence (Table 4).

## 4. Discussion

Regardless of the great efforts made for improving BrC patient management, it remains the most deadly cancer among women [1]. Genomic and expression profiling analysis granted an insight of tumour’s true molecular features and improved respective biology’s understanding [27]. ESMO distinguishes four intrinsic subtypes (luminal A, luminal B, HER-2, and “basal-like”) that display different patterns of gene expression, also presenting different prognoses and clinical outcome [5]. Additionally, the recognition of these entities is currently used for treatment decision making [28]. Indeed, ER, PR, and HER-2 IHC analysis provides an intrinsic classification of tumours identifying, among others, luminal B-like (HER-2+) and HER-2+ (non-luminal) tumours, both characterized by HER-2 receptor overexpression [5]. The standard of care of these HER-2+ subtypes patients’ includes the use of trastuzumab [4,5]. However, recurrence and the development of metastatic disease dampen the effectiveness granted by trastuzumab [29]. Hence, new biomarkers amenable to improve the identification of BrC patients that are most likely to benefit are urgently needed.

ARID1A and ANXA1 expression were suggested to associate with trastuzumab resistance. Specifically, ANXA1 was also implicated in signalling pathways that affect trastuzumab effectiveness [23,30,31]. In parallel, ARID1A loss was associated with a worse prognosis in several tumours [18,19,20], although its function in BrC is not entirely understood [14,15,32]. Furthermore, those studies have only assessed a limited number of HER-2+ tumours samples [32,33]. Indeed, most statistically significant associations of ARID1A and outcome were established for basal-like BrC [34].

Concerning ANXA1, its expression was associated with BrC aggressiveness, progression, higher metastatic potential, poor prognosis, and also triple-negative phenotype [30,35,36,37,38]. Remarkably, ANXA1 was reported to modulate cell adhesion and motility through transforming growth factor-β (TGFβ) activation and, thus, epithelial-mesenchymal transition (EMT) switch, supporting the earlier described BrC features [39,40,41,42]. Additionally, TGFβ also activates PI3K signalling pathway, a mechanism implicated in tumour cells’ unresponsiveness to trastuzumab. Specifically, AKT activation was implicated in such resistance, being associated with worse prognosis in some types of cancer, including BrC [43,44,45,46].

In this study, we evaluated 215 HER-2+ BrC patients’ specimens to investigate the value of ARID1A and ANXA1 expression on clinical outcome and prediction of trastuzumab resistance.

No significant differences were found concerning these proteins’ value as predictors of trastuzumab resistance. Previous studies addressing this issue have used recurrence-free survival as a surrogate definition of resistance to trastuzumab [13]. Nonetheless, this is not the most accurate definition of resistance as patients may experience recurrence many years after receiving trastuzumab. Given that trastuzumab is usually administered for a relatively short period of time (1 year), we considered that recurrence during this period, or in a brief period (for instance, 6 months) after Trastuzumab cessation, more accurately indicates unresponsiveness to trastuzumab. Indeed, another research team considered this time frame for patient inclusion in a clinical trial evaluating the value of another treatment for HER-2+ patients that either recurred or progressed on trastuzumab [26]. Hence, a proper definition of “trastuzumab resistant patients” must be established to standardize future studies regarding prediction biomarkers evaluation.

Contrary to what was reported by Berns et al. [13], an inverse association between ARID1A and ANXA1 was not depicted by our cohort. The authors primarily based their findings on results obtained through functional assays in cell lines. Additionally, they resorted to a The Cancer Genome Atlas (TCGA) panel of BrC patients and found the same association between ARID1A and ANXA1 protein expression. However, this protein analysis differs from ours since they used data from reverse-phase protein array (RPPA) instead of IHC, and the series comprised all subtypes of BrC patients, and not only HER-2+. Furthermore, given that in our study, only 13 cases comprise ARID1A “high intensity” category, this may be accountable for the lack of statistical significance observed.

Moreover, since an inverse correlation between ARID1A and ANXA1 was suggested and one group reported that loss of ARID1A mRNA could be attributed to promoter hypermethylation [47], we also performed quantitative methylation-specific PCR (qMSP). However, no aberrant methylation was found for the same promoter region, in our samples. Nonetheless, to our knowledge, that is the only study reporting that ARID1A downregulation is associated with promoter methylation. The discrepancies observed might result from the different methodologies used, since we performed qMSP and not methylated DNA immunoprecipitation followed by PCR, and the small number of samples included in the study (*n* = 38) by the other research team [47]. Of note, our study only included HER2+ tumours, contrary to others.

Concerning ARID1A expression, our results are in line with the TCGA dataset in which high ARID1A was reported in invasive and mucinous carcinomas, thereby suggesting its involvement in breast carcinogenesis [48]. Nonetheless, other studies refer an association between lower levels and patients’ worse prognosis [14,15,34].

Importantly, HER-2+ (non-luminal) tumours depicted higher ANXA1 expression, which is consistent with previous studies that reported ANXA1 significance in hormone receptor (HR) negative BrC subtypes [39,49]. This suggests that ANXA1’s oncogenic role in BrC may be attenuated in tumours expressing HR, and that its expression may be more relevant in HER-2+ (non-luminal) tumours. Nevertheless, the relatively small size of our cohort implies that further validation is necessary in a larger patient cohort.

Herein, patients with higher ARID1A and ANXA1 expressing tumours showed increased recurrence risk and a higher risk of dying from this disease. Hence, these protein expressions may be useful as recurrence and survival biomarkers. However, it should be considered that, due to the small number of events, ARID1A was not included in the multivariable analysis, thus, further studies with larger cohorts must address this topic. Moreover, since ARID1A and ANXA1 contribute to poorer prognosis and pre-exist in treatment-naïve tumours, they may assist in identifying which HER-2+ patients might require a different therapeutic approach. Since ARID1A and ANXA1 may render trastuzumab resistance through activation of the Pi3K/AKT/mTOR signalling pathway, additional therapeutics targeting this pathway should be considered for this subpopulation of patients. Indeed, other studies attempted to associate AKT activation and response to trastuzumab. A recent study evaluating the effect of carboxyl-terminal modulator protein (CTMP) in trastuzumab resistance, showed that AKT activation is implicated in tumours’ unresponsiveness in HER-2+ BrC patients [45]. Moreover, higher levels of CTMP were related to worse survival in HER-2+ patients. In the same line, by functional assays, higher phosphorylated AKT levels were correlated with resistance to trastuzumab. Hence, AKT signalling or its downstream effectors’ inhibition may also be used as a therapeutic approach to overcome trastuzumab resistance. Moreover, despite these proteins’ role in BrC initiation and progression is far from understood, ANXA1’s ability to discriminate luminal B-like (HER-2+) and HER-2+ (non-luminal) subtypes, contributes to a better patient stratification regarding treatment strategy.

The main limitations of this study were the relatively small number of HER-2+ (non-luminal) tumours and the limited number of recurrences and deaths observed in this patients’ cohort. Importantly, it should be recalled, once again, that current guidelines that specifically define resistance to trastuzumab are still lacking. To overcome that restraint, we have used the definition reported by the EMILIA clinical trial, which focused on the best treatment to be assigned to HER-2+ locally advanced or metastatic BrC patients who stopped responding to trastuzumab [26].

To the best of our knowledge, this is the first study assessing the prognostic and prediction value of ARID1A and ANXA1 proteins’ in HER-2+ BrC patients treated with trastuzumab.

Nonetheless, larger, multicentric, and extended follow-up studies are demanded to validate ANXA1 and, especially, ARID1A value in HER-2+ BrC outcome. Since ANXA1 showed to be a promising prognostic biomarker, it might be interesting to assess its mRNA levels. Nonetheless, a prognostic test based on this gene’s expression would further require a rigorous validation.

Overall, our results support a prognostic value of ANXA1 in HER-2+ BrC patients treated with trastuzumab-based therapy. If standardization and validation are achieved, ANXA1’s assessment will provide a useful clinical asset for patient stratification and prognosis.

## Figures and Tables

**Table 1:** Adenine thymine-rich interactive domain 1A (ARID1A) and annexin A1 (ANXA1) primary antibodies used in immunohistochemistry (IHC) and conditions.

Antibody	Antigen Retrieval Method	Buffer	Dilution	Incubation Time	DAB (min)
**ARID1A** **(sc-32761) Santa Cruz Biotechnology**	Microwave	EDTA	1:800	Overnight at 4 °C	10
**ANXA1 (713400) Invitrogen**	Water bath		1:1500	1 h at room temperature	7

**Table 2:** Clinical and pathological features of luminal-B like (HER-2+) and HER-2+ (non-luminal) breast cancer (BrC) patients enrolled in the study.

Clinicopathological Features	Luminal B-Like (HER-2+)	HER-2+ (Non-Luminal)
**Patients (*n*)**	167	48
**Age median (range)**	51 (24–71)	54.5 (27–69)
**Histological type (%)**		
Invasive Carcinoma, no special type (NST)	128 (76.6)	43 (89.6)
Invasive lobular carcinoma	3 (1.8)	1 (2.1)
Other invasive carcinoma subtypes ^a^	36 (21.7)	4 (8.5)
**Lymphovascular invasion (%)**		
No	88 (52.7)	20 (41.7)
Yes	69 (41.3)	25 (52.1)
Not determined	10 (6)	3 (6.7)
**Grade (%)**		
G1 and G2	84 (50.3)	13 (27.1)
G3	83 (49.7)	34 (70.8)
Not determined	-	1 (2.1)
**Oestrogen Receptor Status (%)**		
Positive	167 (100)	-
Negative	-	48 (100)
**Progesterone Receptor Status (%)**		
Positive	128 (76.6)	-
Negative	39 (23.4)	48 (100)
**Primary tumour (T) (%)**		
T1 & T2	152 (91)	45 (93.8)
T3 & T4	13 (7.8)	3 (6.3)
Not determined	2 (1.2)	
**Regional lymph node (N) (%)**		
N0	67 (40.1)	18 (37.5)
N+	99 (59.3)	30 (62.5)
Not determined	1 (0.6)	-
**Stage (%)**		
I/II	121 (72.5)	37 (77.1)
III	45 (26.9)	11 (22.9)
Not determined	1 (0.6)	-

^a^—Includes medullary, mucinous, and mixed type carcinoma (invasive carcinoma, NST, and micropapillary carcinoma).

**Table 3:** Cox regression models assessing the potential of clinical variables and ANXA1 immunoexpression in the prediction of breast cancer mortality.

Breast Cancer Mortality	Variable	Hazard Ratio (HR)	95% CI for HR	*p*-Value
**Univariable**	Stage		2.415–10.268	<0.001
	I and II	1		
	III	4.980		
	ANXA1		1.259–5.189	0.009
	Negative	1		
	Positive	2.557		
**Multivariable**	Stage		2.374–10.093	<0.001
	I and II	1		
	III	4.895		
	ANXA1		1.309–5.393	0.008
	Negative	1		
	Positive	2.658		

**Table 4:** Cox regression models assessing the potential of clinical variables and ANXA1 immunoexpression in the prediction of relapse.

Relapse	Variable	Hazard Ratio (HR)	95% CI for HR	*p*-Value
**Univariable**	Stage		2.215–7.490	<0.001
	I and II	1		
	III	4.073		
	ANXA1		1.239–4.304	0.008
	Negative	1		
	Positive	2.309		
**Multivariable**	Stage		2.270–7.817	<0.001
	I and II	1		
	III	4.213		
	ANXA1		1.296–4.499	0.005
	Negative	1		
	Positive	2.415		

## Supplementary Materials

The following are available online at https://www.mdpi.com/2077-0383/9/12/3911/s1, Supplementary File 1, which includes: Figure S1—cumulative incidence function plots according to (A) lymphovascular invasion, (B) T stage, (C) N stage, and (D) stage. *p* values obtained by Gray’s test for breast cancer mortality; Figure S2—cumulative incidence function plots according to (A) lymphovascular invasion, (B) T stage, (C) N stage, and (D) stage. *p* values obtained by Gray’s test for breast cancer relapse.
