# Supplementary material for: Clinical Significance of ARID1A and ANXA1 in HER-2 Positive Breast Cancer
Source: J Clin Med. 2020 Dec 2;9(12):3911. doi: 10.3390/jcm9123911 (PMC7761245; doi:10.3390/jcm9123911)
Supplement: Supplementary file 1 [file jcm-09-03911-s001.pdf]

SUPPLEMENTARY FILE 1

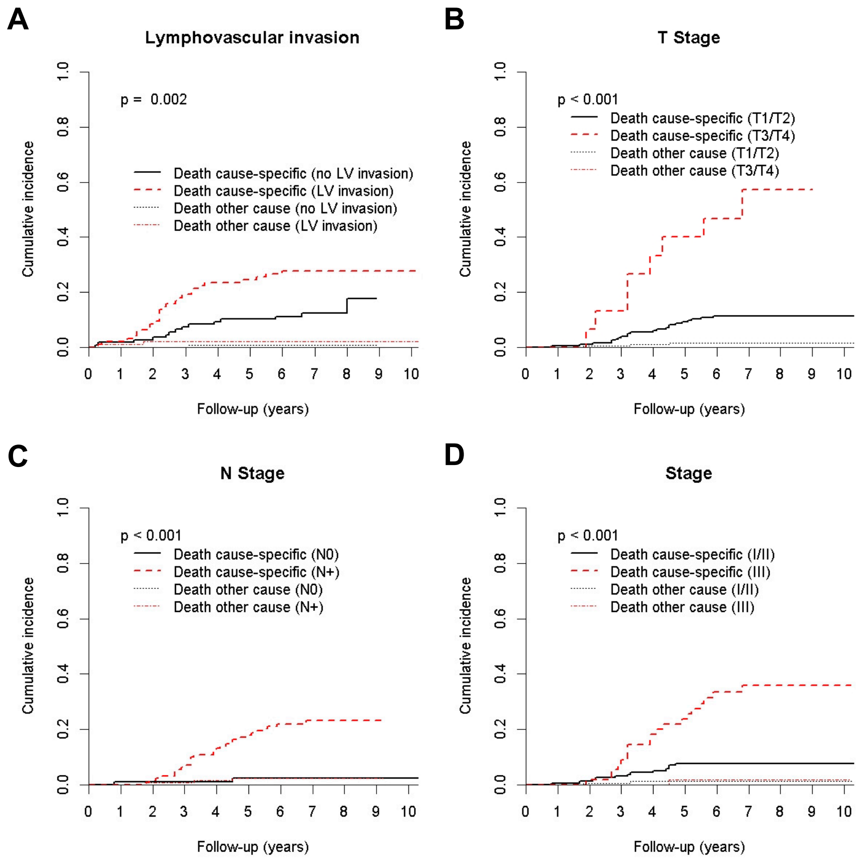

Figure S1—cumulative incidence function plots according to (A) lymphovascular invasion, (B) T stage, (C) N stage, and (D) stage. p values obtained by Gray's test for breast cancer mortality.

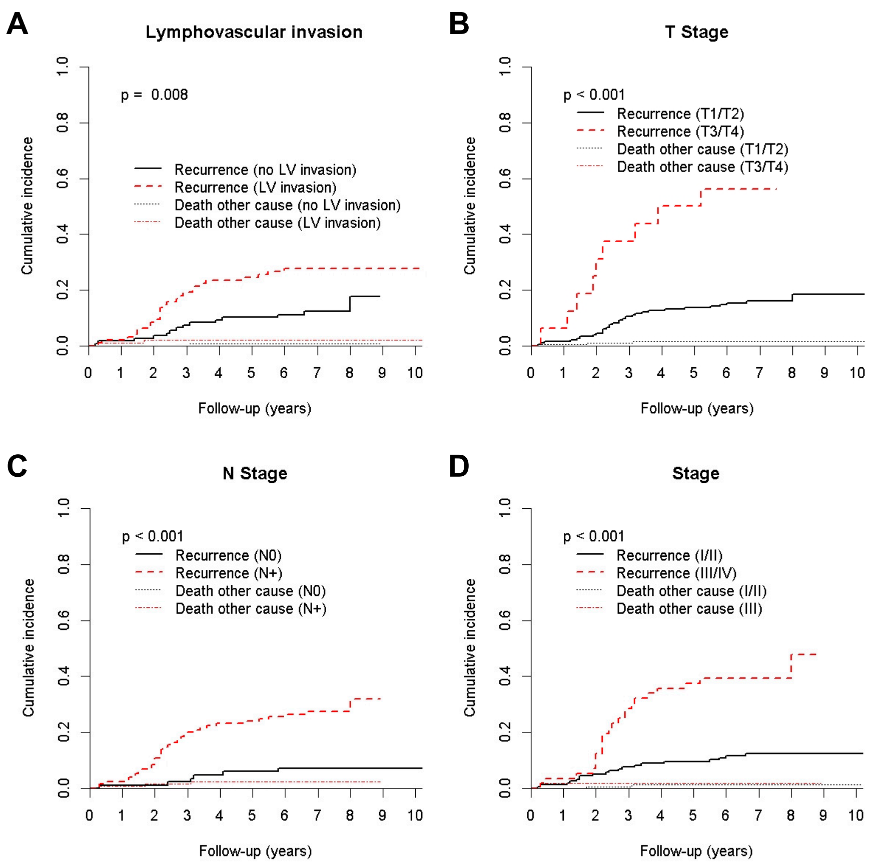

Figure S2—cumulative incidence function plots according to (A) lymphovascular invasion, (B) T stage, (C) N stage, and (D) stage. p values obtained by Gray's test for breast cancer relapse.
